# FBXO22 mediates polyubiquitination and inactivation of LKB1 to promote lung cancer cell growth

**DOI:** 10.1038/s41419-019-1732-9

**Published:** 2019-06-19

**Authors:** Xiao-Na Zhu, Ping He, Liang Zhang, Shuo Yang, Hui-Lin Zhang, Di Zhu, Meng-Di Liu, Yun Yu

**Affiliations:** Key Laboratory of Cell Differentiation and Apoptosis of Chinese Ministry of Education, Rui-Jin hospital, Shanghai Jiao-Tong University School of Medicine (SJTU-SM), Shanghai, China

**Keywords:** Cancer, Non-small-cell lung cancer

## Abstract

Liver kinase B1 (LKB1) regulates both cell growth and energy metabolism. Inactivated mutations of LKB1, observed in 20–30% of nonsmall cell lung cancers (NSCLC), contribute significantly to lung cancer malignancy progression. However, the upstream signalings regulating LKB1 activity remain incompletely understood. Here, we present evidence that FBXO22 interacts with and promotes polyubiquitination of LKB1. More intriguingly, FBXO22 mediates Lys-63-linked LKB1 polyubiquitination and inhibits kinase activity of LKB1. Furthermore, over-expression of FBXO22 promotes NSCLC cell growth through inhibiting LKB1-AMPK-mTOR signaling in vitro and in vivo. Clinically, FBXO22 is highly expressed in human lung adenocarcinoma and high FBXO22 expression predicts significant poor prognosis. Our study provides new insights into the upstream regulation of LKB1 activation and identifies FBXO22 as a potential therapeutic target for lung cancer treatment.

## Introduction

Lung cancer is the leading cause of cancer-related deaths in the world. There were an estimated 228,150 new cases and 142,670 deaths in 2018 within USA^1^. Nonsmall cell lung cancers (NSCLC), accounting for ~85% lung cancers, are often diagnosed at advanced stage and associated with poor prognosis^2^. Recently, in addition to many relevant gain-of-function mutations in oncogenes including Epidermal Growth Factor Receptor (EGFR) and K-RAS^3,4^, loss-of-function mutations in tumor suppressors such as liver kinase B1 (LKB1) are proved to play a pivotal role in NSCLC initiation and progression^5^.

LKB1, also known as STK11, is initially identified as a tumor suppressor gene mutated in patients with inherited cancer disorder Peutz–Jeghers syndrome^6^. Later on, somatic mutations and inactivation in LKB1 have also been observed in 20–30% of NSCLCs^5^. As a major serine/threonine kinase, LKB1 phosphorylates and activates the AMP-activated protein kinase (AMPK), which functions to control NSCLC tumorigenesis and therapeutic response^7–10^. Therefore, the regulation of LKB1 activity would have an important role in the developmemt of LKB1-proficient NSCLCs. A number of studies have described the influence of posttranslational modifications in governing LKB1 activity. For instance, LKB1 undergoes polyubiquitination and deacetylation to maintain its activity and LKB1-STRAD-MO25 complex integrity^11,12^. In addition, LKB1 could be phosphorylated by B-RAF downstream kinase and Aurora kinase A to block its interaction with and function of AMPK^13,14^. Moreover, a critical SUMO1 modification of LKB1 enhances the interaction of LKB1 with AMPK during energy stress^15^. All these findings suggest that posttranslational modifications may have important roles for LKB1 activation. However, the regulation of LKB1 activity in NSCLC is largely unknown.

The F-box protein FBXO22, a not well-recognized protein, contains the 40-amino-acid F-box domain, which binds Skp-1 to create a link to Cullin-1, then form the SCF (Skp-1, Cullin-1, F-box protein) complex to constitute an E3 ligase for ubiquitylation^16^. So far, the biological functions and molecular mechanisms of FBXO22 in lung cancer remain unexplored. In this study, we report that FBXO22 interacts with and targets LKB1 for K63-linked ubiquitination, then in turn inhibits LKB1 activity. Moreover, FBXO22 is highly expressed in lung adenocarcinoma patients, and promotes NSCLC cell growth via inhibiting LKB1/AMPK/mTOR signaling.

## Materials and methods

### Cell lines and cell culture

H322, H446, H460, H661, H1299, and BT549 cells were maintained in RPMI-1640 supplemented with 10% FBS. A549, Hela, MEF, and 293T cells were maintained in DMEM supplemented with 10% FBS. MCF-7 cells were maintained in DMEM supplemented with 10% FBS and 0.01 mg/ml human recombinant insulin. Cells were cultured in a humidified incubator at 37 °C with 5% CO_2_. All of the cell lines were purchased from the cell bank of the Chinese Academy of Sciences (Shanghai, China). For treatment with the NAE1-specific inhibitor MLN4924, cells were treated with 1 μM MLN4924 for 24 h.

### Patients

A chip containing 84 pairs of formaldehyde-fixed, paraffin-embedded tumors and adjacent normal tissues samples from lung cancer patients was purchased from Shanghai Outdo Biotech Co (Shanghai, China) with detailed information described in the Supplementary Table S1. In addition, we collected six pairs of lung cancer and adjacent normal tissue specimens from Rui-Jin Hospital affiliated to Shanghai Jiao-Tong University School of Medicine for analyzing FBXO22 protein expression. These studies were approved by the Medical Ethical Committee of the Rui-Jin Affiliated Hospitals, and informed consent was obtained from all subjects or their relatives.

### Immunohistochemical staining (IHC)

The protein expression levels of FBXO22 were analyzed by IHC with anti-FBXO22 polyclonal antibody. All of the staining was assessed by pathologists who were blinded to the origin of the samples and subject outcome. The intensity of nuclear staining was scored according to the depth of coloration, divided into 0, 1, 2, and 3 fractional grades (no staining = 0; weak staining = 1; moderate staining = 2; strong staining = 3). The positive rate of nuclear staining was scored according to the principle of 0–24% = 1, 25–49% = 2, 50–74% = 3, 75–100% = 4. Finally, the score of the staining intensity and the positive rate were multiplied to obtain the final immunoreactive score of the FBXO22 expression level.

### Plasmids, shRNAs, CRISPR-Cas9s, and transfections

Flag-tagged LKB1, LKB1(1–88), LKB1(1–243), LKB1(1–309), LKB1(310–433), LKB1(Δ89–243), FBXO22, and Culin1 were generated by inserting correspondent CDS into pBabe-3× Flag vector; For HA-tagged wild-type ubiquitin and mutation, correspondent CDS were inserted into PCDNA3.0-HA vector. Plasmids expressing FBXO22 and FBXO22ΔF were generated by inserting correspondent CDS into PLVX-IRES-ZsGreen1 vectors, respectively. His-tagged AMPKα1-312 were generated by inserting correspondent CDS into pET-28a (+) vector. Plasmids for knockdown were constructed by inserting corresponding shRNA sequences into the pSIREN Retro-Q plasmid (clontech). The shRNA sequences specially targeting FBXO22 and LKB1 are, respectively, GTGTGGTCCTTGTCTTTGGTT (shFBXO22#1), CGCATCTTACCACATACAGTT (shFBXO22#2) and CATCTACACTCAGGACTTCAC (shLKB1); CRISPR-Cas9 Plasmids for FBXO22 were Purchases form Obio Technology (Shanghai), The gRNA sequences specially targeting FBXO22 were inserted into pLenti-U6-gRNA-mCMV-SaCas9-P2A-sfGFP, The gRNA sequences are CTTATGGAGGGAGTGTGTGCG(gFBXO22#1), GACCCGCGGAGCACCTTCGTG (gFBXO22#2). For transient transfection, Lipofectamine 2000 transfection reagent (Invitrogen, Carlsbad, CA) was used following the manufacturer’s protocol. For cell transduction, retroviruses or lentiviruses were prepared by transient cotransfection with helper plasmids into 293T cells using X-tremeGene9 (Roche, Basel, Switzerland).

### Immunoprecipitation, immunoblotting, and antibodies

Cells were harvested and lysed with RIPA buffer (50 mM Tris–HCl, pH 7.6; 150 mM NaCl; 1 mM EDTA; 1% NP-40; 1% protease inhibitor cocktail; 1 mM PMSF), centrifuged at 17,000 *g* for 10 min at 4 °C. For immunoprecipitation of Flag-tagged proteins, supernatants were incubated with anti-Flag M2 Affinity Gel (SigmaAldrich) at 4 °C overnight. Otherwise, supernatants were incubated with indicated antibodies overnight and protein A/G-agarose beads (Santa Cruz, CA) for 4 h at 4 °C. The precipitates were washed three times with PBST buffer (PBS with 0.1% Tween-20), boiled in SDS-sample buffer, and subjected to immunoblotting analysis. For the protein expression analysis, standard western blotting was carried out with the following antibodies used: LKB1 (#3050), AMPKα (#2532), P-AMPKα (#2535), Raptor (#2280), P-Raptor (#2083), ACC (#3662), P-ACC (#11818), SKP1 (#12248), P-P70S6K (#9234), P70S6K (#2708), MO25α (#2716) were purchased form Cell Signaling Technology; FBXO22 (13606-1-AP) was purchased from Proteintech Group; Ubiquitin-K63 (EPR8590-448), NEDD8 (ab81264) were purchased from Abcam Technology; HA (H6908), Flag (A8592) and β-actin (A5316) were purchased from Sigma-Aldrich.

### In vitro kinase assay

Recombinant His-AMPKα_1–312_ protein was expressed in BL21 bacteria and purified from the bacterial lysates by nickel-agarose column. Endogenous LKB1 was IP from cells by anti-LKB1 antibody. Then immunoprecipitates were incubated with recombinant His-AMPKα_1–312_ for 30 min at 30 °C in 50 μl of reaction buffer (Kinase buffer with 0.5 mM ATP purchased from Cell Signaling Technology). After incubation, proteins were boiled in SDS-sample buffer and subjected to immunoblotting analysis. The kinase activity of LKB1 was directly determined by measuring Thr172 phosphorylation of recombinant AMPKα_1–312_ using anti-phospho-AMPKα (Thr172) antibody.

### Denaturing ubiquitination assay

Cells were harvested and lysed with 70 μl 1× SDS lysis buffer by boiling at 100 °C for 20 min, then centrifuged at 17,000 *g* for 10 min at 4 °C. The supernatants were diluted by RIPA buffer and suffered to immunoprecipitation of Flag-tagged proteins as described earlier. The precipitates were washed three times with PBST buffer and boiled in SDS-sample buffer, then subjected to immunoblotting analysis.

### Cell proliferation and colony formation assay

Cell proliferation was evaluated by the speed of cell growth. In brief, cells were digested into single cell suspension and planted in the six-well plate with 1.5 × 10^5^ in complete growth media for cell proliferation by counting every 2 days. For colony formation assay, 200 cells were planted in six-well plate and allowed to grow until visible colonies formed, about two weeks later, Cell colonies were fixed with cold methanol, stained with 0.1% crystal violet for 30 min, washed, air dried, photographed, and counted.

### Tumor xenograft experiment

A total of 3 × 10^6^ cells were 1:1 mixed with matrigel (Corning, 354248) in a total volume of 150 μl. The mixture was subcutaneously injected into the dorsa of nude mice (6 weeks old female; Shanghai SLAC Laboratory Anima). The tumor growth was measured every 3 days for 6 times using a digital caliper. The tumor volume was determined by the length (a) and width (b) as *V* = *ab*^2^/2. Finally, the tumors were isolated and weighted after mice sacrificed via cervical vertebral dislocation.

### Statistical analysis

The statistical analysis between normal and LUAD or LUSC groups by unpaired two-tailed student’s *t* test. Overall survival (OS) was calculated using Kaplan–Meier method. The survival distributions were compared through log-rank test by SPSS 16.0 software (Chicago, IL, USA), the data between two growth curves of tumor were examined by repeated measures analysis of variance, other *P*-values for comparison between line-linked groups were obtained by student’s *t* test. All statistical tests were two-sided, and *P* < 0.05 was statistically significant. Statistical analyses were conducted using SPSS 16.0 software.

## Results

### FBXO22 is highly expressed in human lung cancer and its expression is a significant prognostic predictor for survival of lung cancer patients

**Fig. 1:**
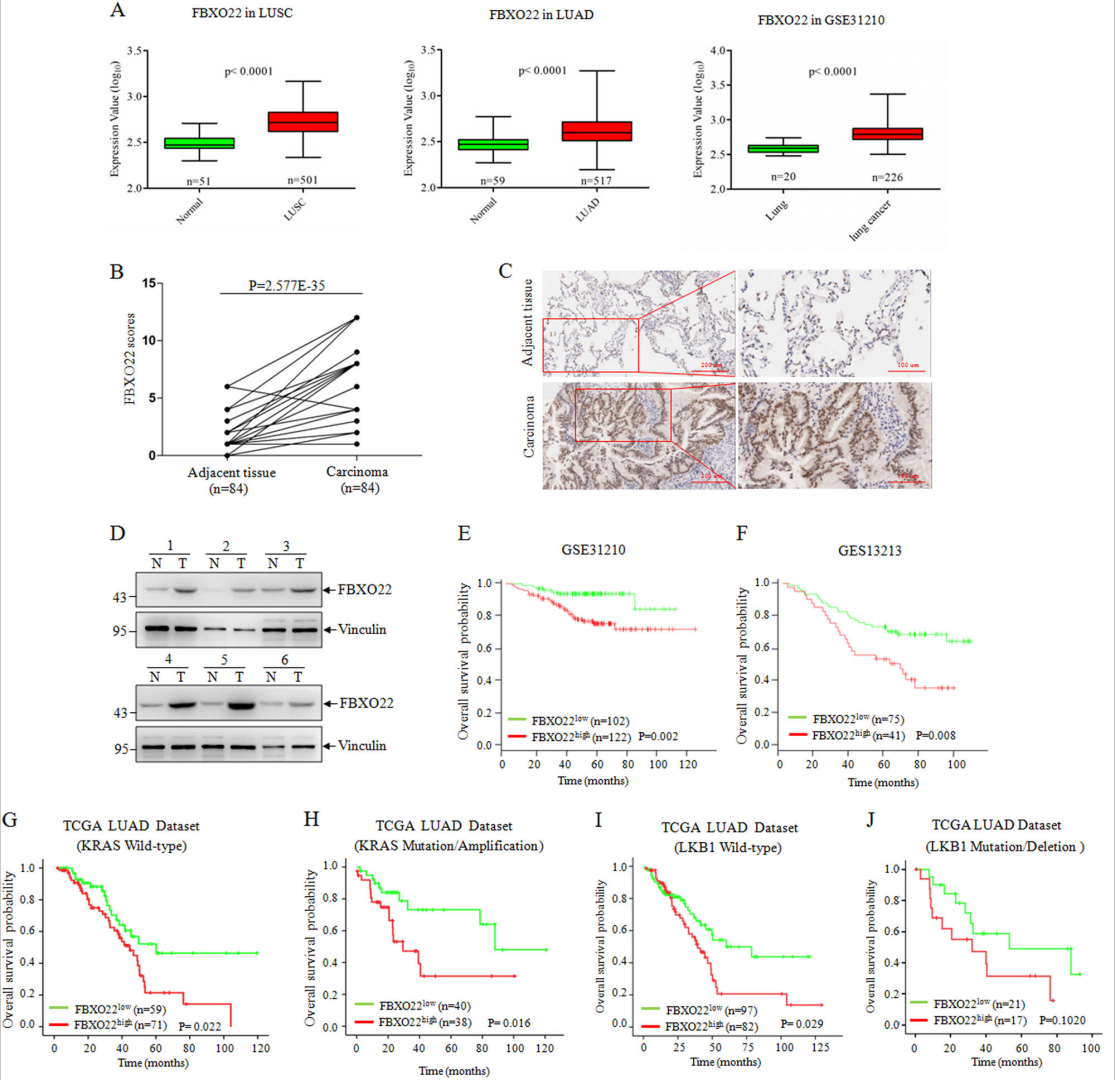
FBXO22 is highly expressed and predicts a poor prognosis in lung cancer. **a** FBXO22 mRNA expression of tumor and normal tissues in TCGA lung squamous cell carcinoma (LUSC) dataset (left), lung adenocarcinoma (LUAD) dataset (medium) and in GSE31210 microarray dataset (right). **b** FBXO22 expression was plotted using the immunohistochemical scores as described in the “Material and methods”. We compared lung cancer tumors with matched adjacent normal tissues using the Mann–Whitney test. **c** Representative images of FBXO22 immunohistochemical staining from one pair of lung cancer tissue and adjacent normal tissue, with scale bars representing 200 μm (left) and 100 μm (right). **d** Expression of FBXO22 in six pairs of clinical lung cancer specimens. N and T mean adjacent normal tissue and paired lung cancer tissue, respectively. **e**, **f** Kaplan–Meier estimates of overall survival of subjects with lung cancers of high and low FBXO22 expression in GSE31210 and GSE13213 lung cancer dataset. Kaplan–Meier estimates of overall survival of subjects with Lung cancers of high and low FBXO22 expression in TCGA lung Adenocarcinoma dataset with wild-type KRAS expression (**g**), mutation/amplification KRAS expression (**h**), wild-type LKB1 expression (**i**) and mutation/deletion LKB1 expression (**j**)

To explore the biological function of FBXO22 in lung cancer, we initially sought to examine FBXO22 expression in clinical samples. First, we found FBXO22 mRNA was highly expressed in both lung squamous cell carcinoma and lung adenocarcinoma in The Cancer Genome Atlas (TCGA) database and the cohort of GSE31210 lung cancer patients^17^ (Fig. 1a). Next, we performed IHC staining on 84 lung adenocarcinoma tissues and the matched adjacent normal tissues, and found that lung adenocarcinoma tissues presented higher FBXO22 expression compared with adjacent normal tissues (Fig. 1b, c). Moreover, an increase of FBXO22 protein level in tumor tissues over adjacent normal tissues was also confirmed by western blot analysis in six paired clinical lung cancer specimens (Fig. 1d). These data indicate that FBXO22 is highly expressed in human lung cancer at the protein level.

Then we also examined the relationship between FBXO22 mRNA expression and patients outcome using two different cohorts of lung cancer patients^17,18^. Kaplan–Meier analysis revealed that highly expressed FBXO22 showed significantly poor OS (Fig. 1e, f). Because the loss-of-function mutations of LKB1 and the gain-of-function mutations of K-RAS are two major lung cancer-driven genes, we further evaluated whether FBXO22 expression predicts prognosis related to the status of LKB1and K-RAS in lung adenocarcinoma patients. The TCGA dataset analysis showed that the negative correlation between FBXO22 expression and OS could not be found in patients with mutation or deletion LKB1 expression (Fig. 1g–j), suggesting the potential link of FBXO22 and LKB1 in lung cancer.

### FBXO22 interacts with LKB1

**Fig. 2:**
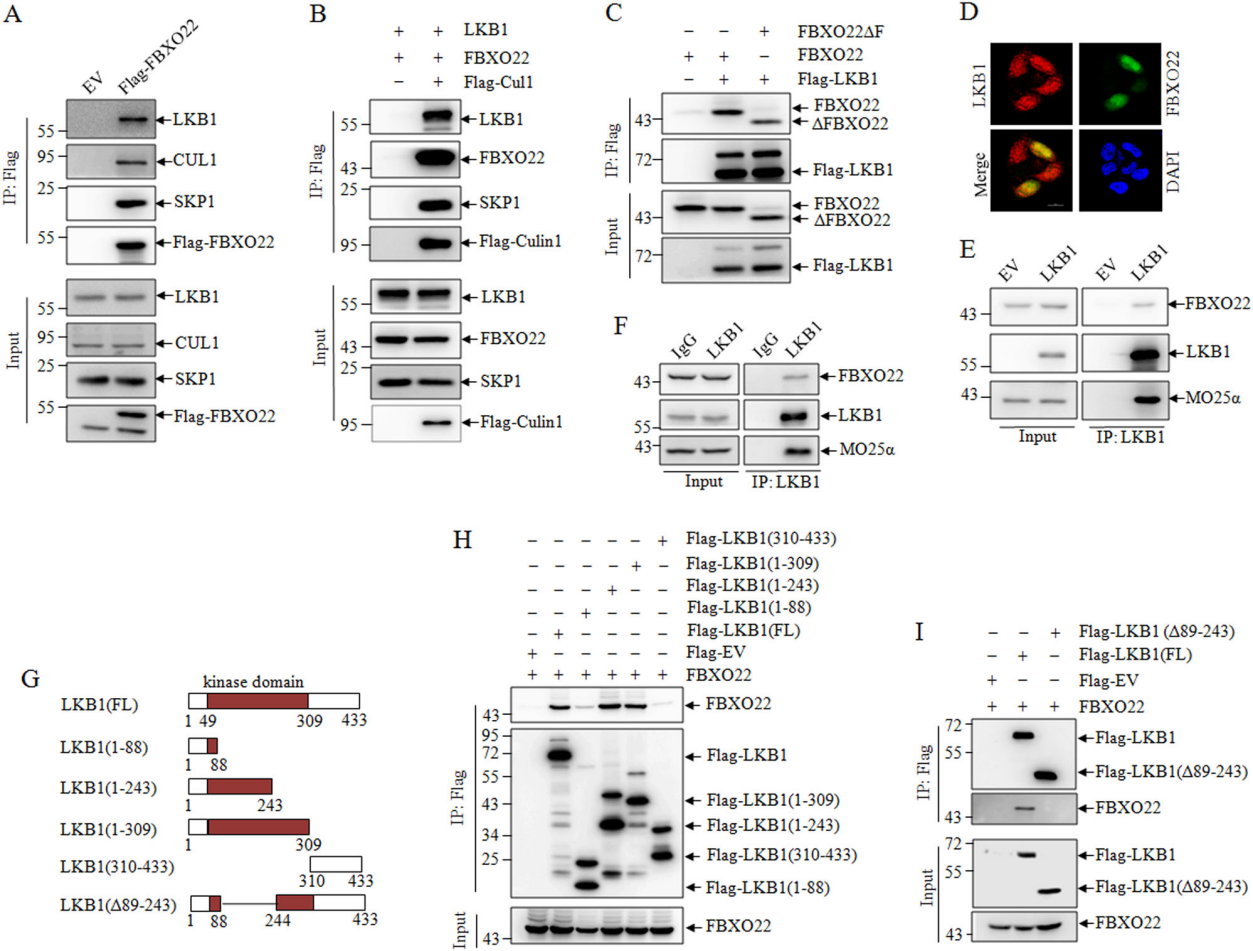
FBXO22 interacts with LKB1. 293T cells were transfected with Flag-FBXO22 (**a**), or FBXO22, LKB1, and Flag-tagged Culin1 (**b**), or Flag-tagged LKB1, FBXO22, and F-box domain deleted mutant FBXO22ΔF (**c**) with corresponding empty vector. Co-IP was performed with M2 beads followed by western blots for the indicated proteins. **d** LKB1-deficient Hela cells were transfected with Flag-tagged LKB1 or GFP-FBXO22 with corresponding empty vector. Immunofluorescence staining of LKB1 and re-staining of DAPI were performed. Scale bar represents 20 μm. **e** LKB1-deficient A549 cells with stable restoration of Flag-LKB1 were immunoprecipitated with anti-LKB1 antibody, and precipitates were detected by western blots. **f** LKB1-proficient H1299 cells were immunoprecipitated with anti-LKB1 antibody, and precipitates were detected by western blots. **g** Schematic illustrations of LKB1 full length (FL), fragments and deleted mutant. Flag-LKB1-FL, Flag-LKB1 fragment (**h**) or Flag-LKB1 deleted mutant (**i**) were transfected into 293T cells together with FBXO22, followed by co-IP with M2 beads, and precipitates were detected by western blots

The above observations led us to detect the direct interaction between FBXO22 and LKB1. For this purpose, HEK293T cells were transient transfected with empty and Flag-FBXO22 expressing plasmids, and cell lysates were immunoprecipitated (IP) by antiFlag antibody. As we expected, LKB1 could be precipitated by ectopically expressed Flag-FBXO22, and also associated with CUL1 and SKP1, core subunits of SCF scaffold, supporting the specificity and effectiveness of the IP assay (Fig. 2a). Furthermore, ectopically expressed Flag-CUL1 could pull down ectopically expressed FBXO22, LKB1, and endogenous SKP1, suggesting that LKB1 interacts with components of SCF^FBXO22^ complex (Fig. 2b). FBXO22 contains an N-terminal F-box domain responsible for binding SKP1 and a C-terminal domain^16^. As depicted in Fig. 2c, the exogenous F-box domain deleted mutant ΔFBXO22 associated with Flag-LKB1 to an extent similar to that found with exogenous full-length FBXO22, proposing the C-terminal domain of FBXO22 is required for its interaction with LKB1 protein. In addition, the GFP-FBXO22 and Flag-LKB1 were colocalized in the nuclear in LKB1-deficient Hela cells (Fig. 2d). Next, we also found the endogenous interaction between FBXO22 and LKB1 in LKB1-deficient A549 (Fig. 2e) and LKB1-proficient H1299 lung cancer cells (Fig. 2f).

To define the FBXO22-binding domain of LKB1, the Flag-tagged LKB1 (FL), and its several fragments, including LKB1 (1–88), LKB1 (1–243), LKB1 (1–309), LKB1 (310–433) (Fig. 2g), were respectively cotransfected with FBXO22 in 293T cells, and the co-IP assay revealed that LKB1 (1–243) and LKB1 (1–309), but not LKB1 (1–88) and LKB1 (310–433) appeared to be able to bind FBXO22 as well as full-length of LKB1 (Fig. 2h). Therefore, we concluded that 89–243 of protein kinase domain of LKB1 was essential for its interaction with FBXO22. To further confirm it, we co-transfected Flag-tagged 89–243 deleted mutant LKB1 (Δ89–243) and LKB1 (FL) with FBXO22, in accordance with the results in Fig. 2i, LKB1 (Δ89–243) could not associate with FBXO22. Collectively, our data suggest that FBXO22 could interact with LKB1, and the 89–243 of protein kinase domain of LKB1 is required for its interaction with FBXO22 C-terminal.

### FBXO22-SCF Ubiquitinates LKB1 via K63-Linkage

**Fig. 3:**
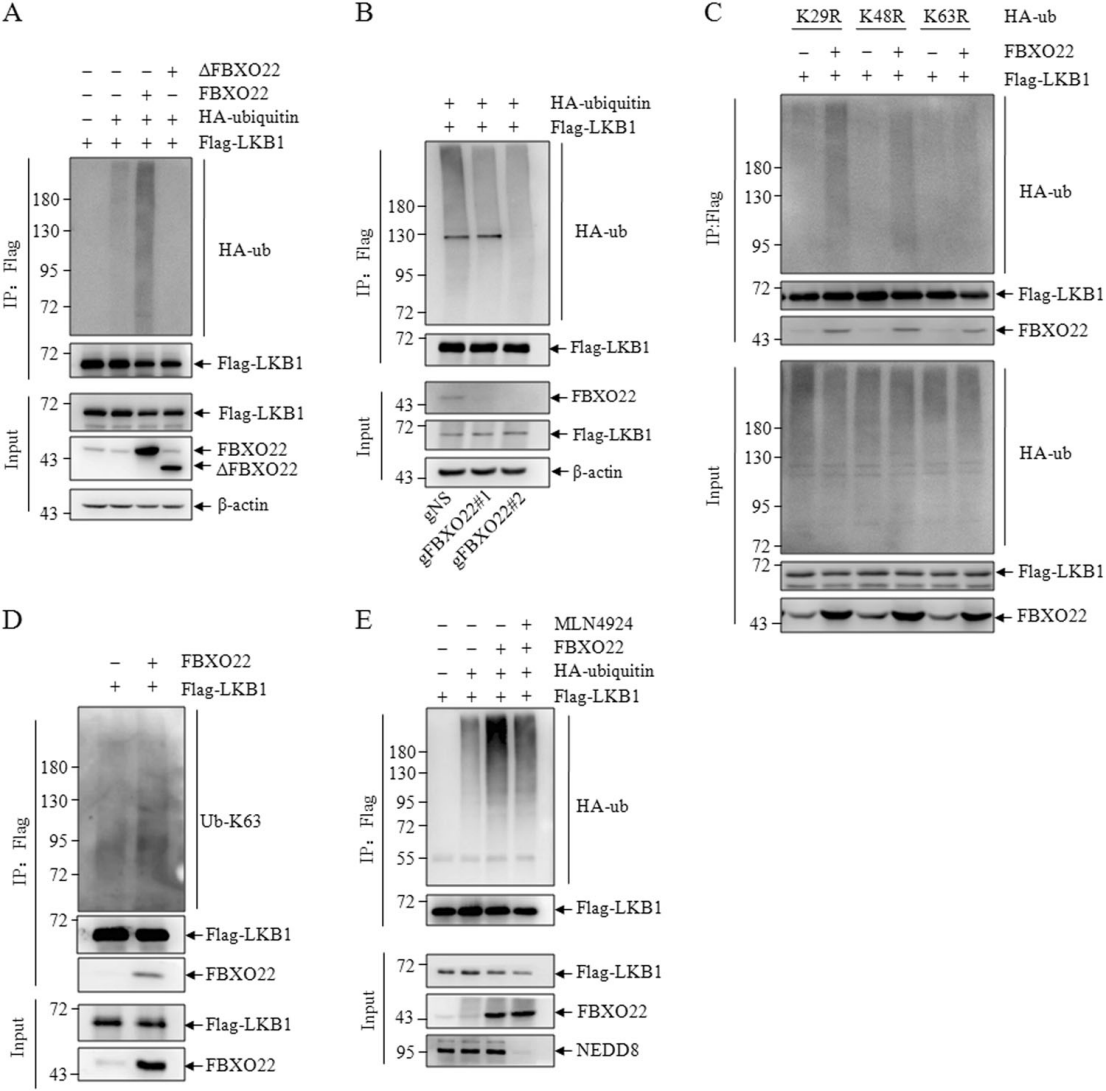
FBXO22 Regulates the polyubiquitination of LKB1. 293T cells were transfected with Flag-tagged LKB1, HA-tagged ubiquitin, FBXO22, and ΔFBXO22 with corresponding empty vector (**a**), or 293T cells with negative control (gNS) or FBXO22 knockdown (gFBXO22#1/ gFBXO22#2) were transfected with Flag-tagged LKB1, HA-tagged ubiquitin (**b**), or 293T cells were transfected with flag-tagged LKB1, HA-tagged ubiquitin mutants and FBXO22 with corresponding empty vector (**c**), or 293T cells were transfected with Flag-tagged LKB1 and FBXO22 with corresponding empty vector (**d**), or 293T cells were transfected with Flag-tagged LKB1, HA-tagged ubiquitin and FBXO22 with or without MLN4924 treatment (**e**), then subjected to denatured co-IP with M2 beads followed by western blots

Because FBXO22 is a substrate recognition component of the SCF complex, our finding that FBXO22 interacts with LKB1 raises the question of whether FBXO22 can promote LKB1 ubiquitination. Using in vivo ubiquitination assays, a strong ubiquitylation signal was detected in the presence of wild-type FBXO22, but not its mutants lacking the F-box domain under denatured IP conditions (Fig. 3a). Consistently, FBXO22 knockdown reduced LKB1 polyubiquitination (Fig. 3b). Polyubiquitin chains linked through seven lysine residues (K6, K11, K27, K29, K33, K48, and K63) of ubiquitin may exert both proteasome-dependent and independent functions. K48-linked polyubiquitin chains are the major targeting signal for proteasomal degradation, whereas K63-linked polyubiquitin chains have nonproteolytic functions in different cellular processes, including kinase activation, DNA repair, and protein trafficking^19^. In order to examine the chain linkage requirements for LKB1 ubiquitination, we applied three mutant forms of ubiquitin to our in vivo ubiquitination assay: Ub-K29R, Ub-K48R, and Ub-K63R, which exclusively eliminate K29, K48, and K63-linked polyubiquitination, respectively. Strikingly, we found that Ub-K63R, but not Ub-K48R and Ub-K29R, blocked FBXO22-mediated polyubiquitination of LKB1 (Fig. 3c). Furthermore, we examined the intrinsic K63 ubiquitination level and found that FBXO22 transfection markedly elevated the K63 polyubiquitination of LKB1 (Fig. 3d). The activity of SCF complex is stimulated by linkage to cullin of the ubiquitin-like protein Nedd8. MLN4924, a specific small molecule inhibitor, specifically blocks the activity of NEDD8 E1 activating enzyme, efficiently inhibits neddylation of cullins, resulting in inactivation of SCF complex. We did in vivo ubiquitination assays with or without MLN4924 treatment, and found that FBXO22 markedly elevated the polyubiquitination of LKB1, which was attenuated by treatment of MLN4924 (Fig. 3e). The results suggested that FBXO22-mediated polyubiquitination of LKB1 requires neddylation of Cullin.

### FBXO22 mediates nondegradative polyubiquitination of LKB1

**Fig. 4:**
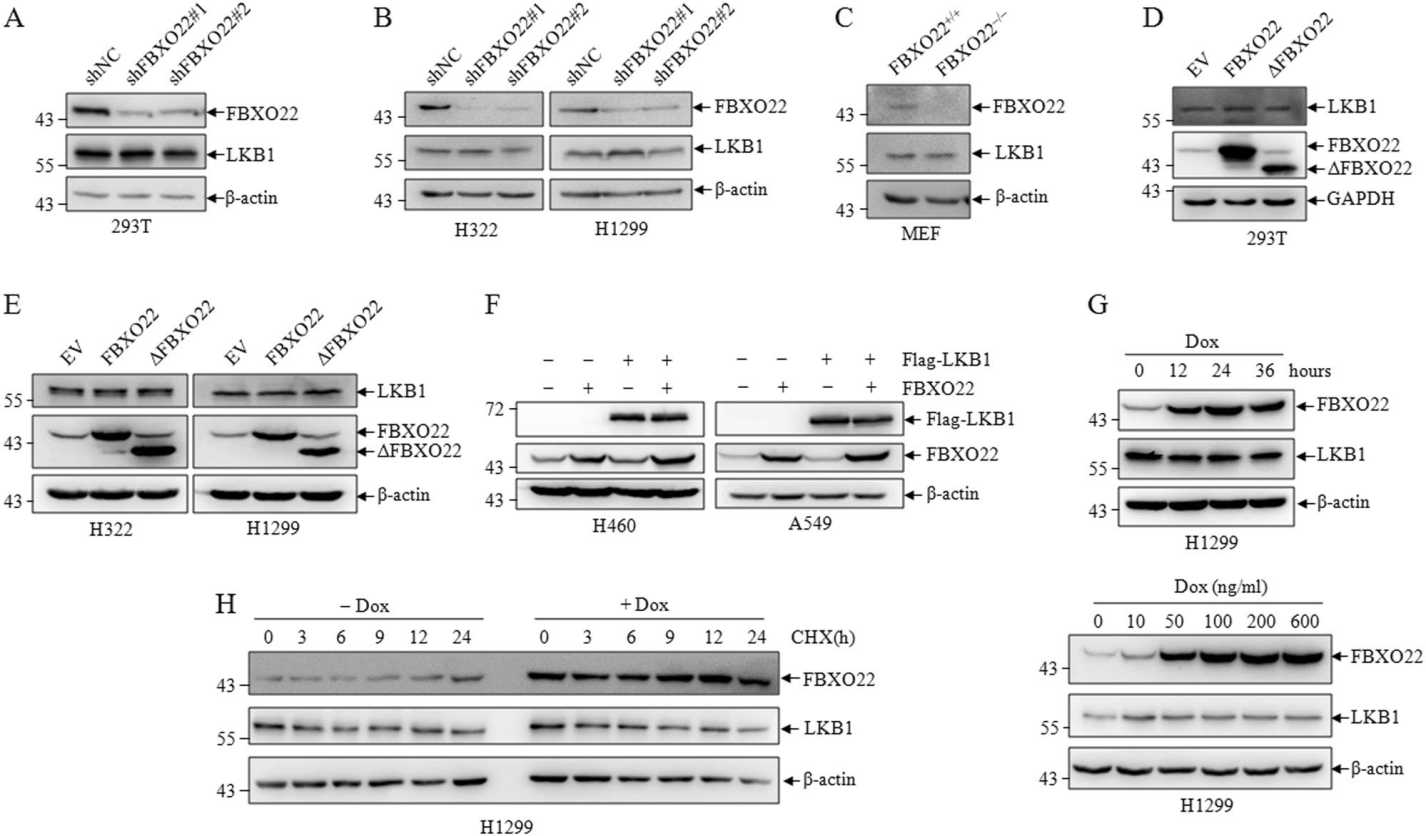
FBXO22 mediates non-degradative polyubiquitination of LKB1. 293T (**a**), H322, and H1299 (**b**) cells were infected with shNC or shFBXO22, cell lysates were analyzed by western blots for the indicated proteins. **c** Cell lysates from wild-type and FBXO22 knockout mice MEF cells were subjected to western blots to detect indicated proteins. 293T (**d**), H322 and H1299 (**e**) cells were transfected with FBXO22 and ΔFBXO22, cell lysates were analyzed by western blots for the indicated proteins. **f** LKB1-deficient H460 and A549 cells were transfected with Flag-LKB1 and FBXO22, cell lysates were analyzed by western blots for the indicated proteins. H1299 cells expressing the Doxycycline (Dox) inducible FBXO22 were treated with Dox for different time and concentration (**g**), the same cells were treated with or without Dox (200 ng/ml) for different time (**h**), cell lysates were analyzed by western blots for the indicated proteins

To study the relationship between FBXO22 and LKB1, two pairs of shRNAs (shFBXO22#1 and shFBXO22#2) specifically against FBXO22 were generated to knockdown FBXO22 along with a control shRNA (shNC) in 293T, H322, and H1299, we did not observe significant changes in LKB1 protein level upon FBXO22 knockdown in these cells (Fig. 4a, b) and other cancer cells (Fig. S1A). Consistently, LKB1 expression was not changed in FBXO22 knockout MEF cells (Fig. 4c). In contrast, over-expression of wild-type FBXO22 and F-box domain deleted mutant ΔFBXO22 also did not affect endogenous LKB1 protein in these cell lines (Fig. 4d, e). In addition, we co-transfected LKB1 and FBXO22 in LKB1 null H460 and A549 lung cancer cells, as shown in Fig. 4f, over-expression of FBXO22 could not change the enforced LKB1 expression. The same results were obtained in two other LKB1 deficient H446 and Hela cancer cells (Figure S1B). Then, we induced FBXO22 expression using doxycycline at different time course or different drug concentration in several tumor cells, the induced FBXO22 expression also had no effect on LKB1 level (Figs. 4g and S1C). To further evaluate the data, we induced FBXO22 expression and examined the protein level at different time points in the presence of cycloheximide, an inhibitor of protein synthesis. Versus the control, over-expression of FBXO22 did not affect the half-life of endogenous LKB1 (Fig. 4h).

### FBXO22 suppresses the kinase activity of LKB1

**Fig. 5:**
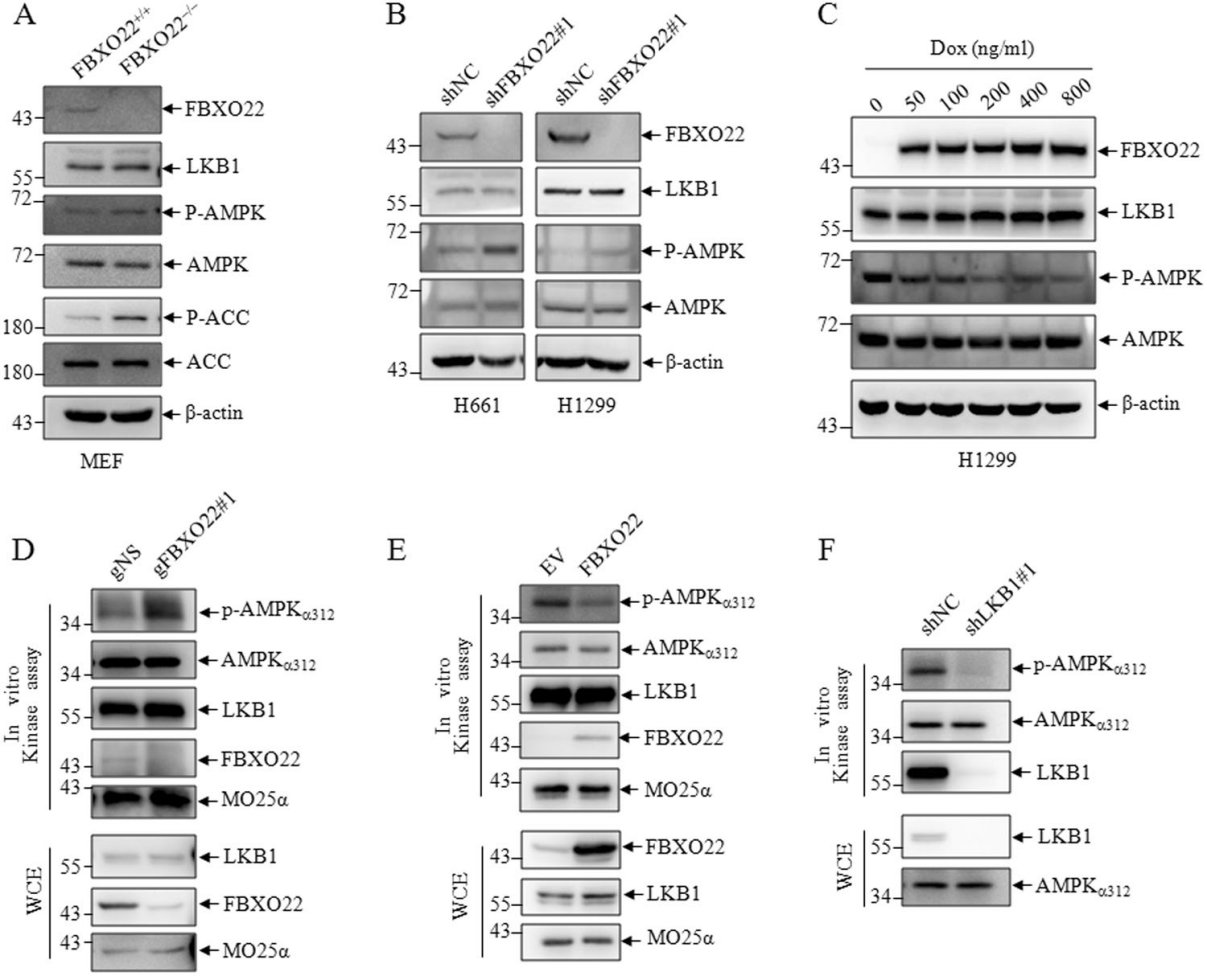
FBXO22 suppresses the kinase activity of LKB1. **a** MEF cells from wild-type and FBXO22 knockout mice subjected to western blots to detect indicated proteins. **b** H661 and H1299 cells were transfected with shNC or shFBXO22#1, cell lysates were analyzed by western blots for the indicated proteins. **c** H1299 cells expressing the Doxycycline (Dox) inducible FBXO22 were treated with Dox for different concentration for 24 h, cell lysates were analyzed by western blots for the indicated proteins. Cells lysates from 293T cells with negative control (gNS) and FBXO22 knockdown (gFBXO22#1) (**d**), or 293T cells with empty vector and FBXO22 over-expression (**e**) or 293T cells with negative control (shNC) and LKB1 knockdown (shLKB1) (**f**) were subjected to immunoprecipitates by anti-LKB1 antibody followed by in vitro LKB1 kinase assay, the precipitates were detected by western blots

Since FBXO22 mediates nondegradative polyubiquitination of LKB1, we investigated whether FBXO22 is involved in regulating the kinase activity of LKB1. We first examined the intrinsic activation of LKB1/AMPK signaling in MEF and *LKB1-proficient* H661 and H1299 lung cancer cells, phosphorylation of AMPKα at Thr172, a well-known indicator of AMPKα activation, was analyzed. The results showed that the phosphorylation of AMPKα was induced in FBXO22 knockout MEF cells (Fig. 5a), and knockdown of FBXO22 increased while induced FBXO22 expression decreased AMPK phosphorylation in *LKB1-proficient* lung cancer cells (Fig. 5b, c), suggesting FBXO22 is critical for maintaining LKB1 activity. To consolidate the data, we next measured LKB1 kinase activity toward AMPKα in vitro. Endogenous LKB1 IP from cells was incubated with recombinant His-AMPKα_1–312_ for kinase reaction. LKB1 kinase activity was determined by measuring Thr172 phosphorylation of recombinant AMPKα_1–312_. Consistent with the previous findings, LKB1 protein isolated from cells with FBXO22 knockdown using CRISP/Cas9 technology displayed an increased ability to phosphorylate AMPKα (Fig. 5d). Vice versa, a reduced AMPKα_1–312_ phosphorylation was shown in FBXO22 over-expression cells (Fig. 5e). Here, LKB1 derived from LKB1 knockdown cells dramatically reduced AMPKα_1–312_ phosphorylation as a positive control (Fig. 5f). These results indicated that FBXO22 impaired the ability of LKB1 to activate AMPK, thereby downregulating the LKB1/AMPK signaling.

### FBXO22 promotes lung cancer cell growth via inhibiting LKB1/AMPK/mTOR signaling

**Fig. 6:**
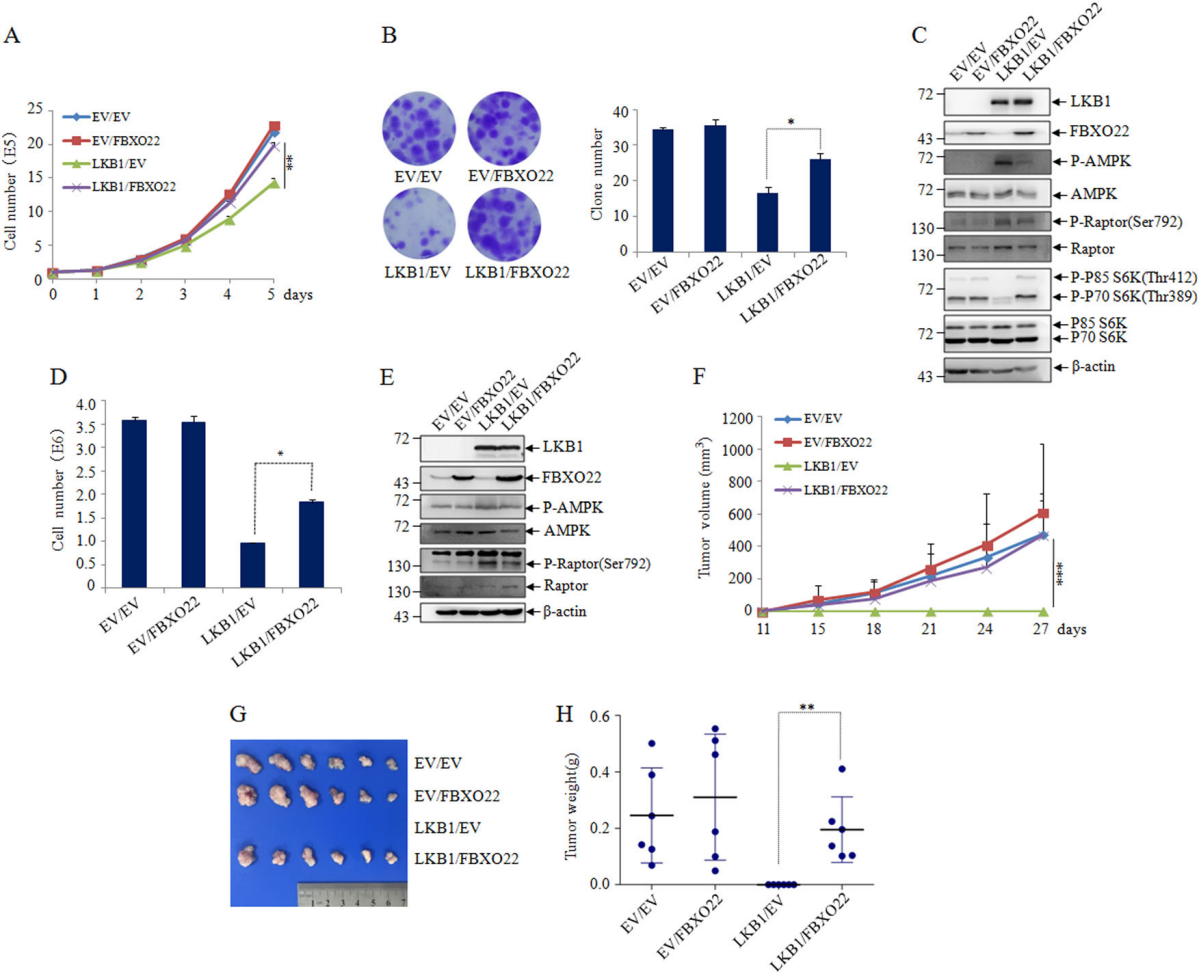
FBXO22 targets LKB1 to accelerate cell growth. **a** Cell counting of EV/EV, EV/FBXO22, LKB1/EV, LKB1/FBXO22 co-transfected A549 cells after 0–5 days of growth. Data are presented as mean ± S.D and significance is **P < 0.01, which was repeated for more than three times. **b** Colony formation capacity of cells discribed as (**a**) was assessed by the number of clones. Representative images were presented (left) and the number of clones was calculated for each well of six-well plates and shown (right). Data are presented as mean ± SD and significance is *P < 0.05, which was repeated for more than three times. **c** Cell lysates from the cells discribed as (**a**) were subjected to western blot to detect the indicated proteins, with β-actin as a loading control. **d** Cell counting of EV/EV, EV/FBXO22, LKB1/EV, LKB1/FBXO22 co-transfected H460 cells after 4 day growth (left). Data are presented as mean ± SD and significance is *P < 0.05, which was repeated for more than three times. **e** Cell lysates from the cells discribed as (**d**) were subjected to western blot to detect the indicated proteins, with β-actin as a loading control. **f**–**h** The cells discribed as (**d**) were subcutaneously injected into nude mice. At least six tumors per condition were analyzed. **f** The tumor growth curve of tumor volume was drawn according the times indicated. Data are presented as mean ± SD. and symbol *** indicates *p* < 0.001 between lined groups. The macroscopic appearances of tumors (**g**) and the tumor weight (**h**) were shown at one month after injection. Data are presented as mean ± SD and symbol ** indicates *p* < 0.01 between lined groups

Activation of the LKB1/AMPK pathway plays an important role in cell growth control^20^. We thus investigated whether FBXO22-mediated inhibition of LKB1 activity regulates lung cancer cell proliferation. For this purpose, LKB1 and FBXO22 were separately transfected or cotransfected into LKB1 null A549 and H460 lung cancer cells, and cell growth of these cells was measured by cell counting and colony formation assay. Our results showed that reintroduction of LKB1 into LKB1-deficient lung cancer cells resulted in cell growth arrest, which was consistent with the previous report^21^. Intriguingly, the ectopically expressed FBXO22 did not cause apparent change in cell proliferation in LKB1-deficient lung cancer cells, but could dramatically rescue LKB1-mediate cell growth inhibition in LKB1 expressed lung cancer cells (Fig. 6a, b, d).

To further evaluate the effect of FBXO22 on LKB1-mediated tumor growth inhibition in vivo, H460 cells infected with EV, LKB1, FBXO22 or LKB1/FBXO22 were injected subcutaneously into two bilateral sites of BALB/c nude mice. In agreement with the in vitro findings, there were no significant difference between tumors generated from EV and FBXO22 infected H460 cells, but the average volume and weight of tumors generated from LKB1/FBXO22-coinfected H460 cells were markedly increased compared to LKB1-infected H460 cells (Fig. 6f–h). Similar results were observed in another LKB1 null Hela cells (Figure S2A-D).

It is reported that one of the major growth regulatory pathways controlled by LKB1/AMPK signaling is through suppression of the mTORC1 signaling achieved through dual phosphorylation of TSC2 and Raptor^22–24^. To ask whether FBXO22 promotes cancer cell growth through LKB1/AMPK/mTOR pathway, we performed further analysis of downstream signaling pathways using p-AMPK(Thr^172^), p-Raptor(Ser^792^) and p-P70S6K(Thr^389^), as indicators of mTOR activation. Western blots showed that compared with LKB1 expression alone in A549 and H460 cells, FBXO22 co-expression in these cells led to decreased phosphorylation of AMPK and Raptor, increased phosphorylation of P70S6K (Fig. 6c, e), suggesting that FBXO22 modulates LKB1/AMPK/mTOR signaling. Taken together, all these data propose that FBXO22 accelerates lung cancer cell growth through inhibiting LKB1/AMPK/mTOR signaling.

## Discussion

Metabolic sensor AMPK signaling plays a central role in the regulation of cell metabolism, survival and proliferation under energy stress, which has been proved as a hallmark of cancer^20,25,26^. LKB1 is the major upstream kinase phosphorylating AMPK, which has been well-defined as a tumor suppressor, since its loss-of-function mutations are commonly detected and directly induce the oncogenesis in some malignancies, especially in NSCLCs^27–31^. Therefore, targeting of LKB1 activity is an attractive strategy which contributes to NSCLC tumorigenesis. In this study, we uncover that an E3 ligase FBXO22 could directly interact with and promote polyubiquitination of LKB1 and show that 89–243 of protein kinase domain of LKB1 is essential for its interaction with FBXO22 C-terminal. Further, FBXO22-mediated LKB1 polyubiquitination primarily occurs through nondegradative K63-linked ubiquitination for non-proteolytic regulation, such as kinase activation. In line with this, we show that FBXO22 inhibits LKB1 activity rather than stability, although the ubiquitinating site(s) on LKB where FBXO22 mediates polyubiquitination take place remains to be identified. It is well-recognized that LKB1 activation is mediated by the assembling of LKB1/STRAD/MO25 complex and subcellular localization of LKB1^32–35^, hence, how the polyubiquitination of LKB1 by FBXO22 interferes LKB1 nuclear translocation or disrupt the binding of LKB1 with STRAD or MO25 still needs to be investigated.

FBXO22 is a newly-identified F-box E3 ubiquitin ligase as a p53-targeting gene^36^. Several proteins have been reported as targets of FBXO22 for proteasomal degradation^37–40^. Our results identified LKB1 as a novel nonproteolytic substrate for FBXO22. First, LKB1 protein level do not change in the condition of FBXO22 knockdown or over-expression in cancer cell lines. Second, FBXO22 induces K63-linked polyubiquitination of LKB1, which mediates nonproteolytic functions instead of proteasomal degradation. Next, we use in vitro kinase assay to demonstrate that FBXO22 suppresses the kinase activity of LKB1. The effect of FBXO22 on LKB1 is partly similar to that of SKP2, also named FBXL1, which is another well-defined F-box protein that also promotes K63-linked polyubiquitination of LKB1 in sites K41, 44, 48, 62 and 64 on LKB1^11^. In our experiments, we identified that these lysines were not required for LKB1 ubiquitination mediated by FBXO22 (Figure S3), which suggests FBXO22 inhibits LKB1 activity while SKP2 maintains LKB1 activity by regulating different ubiquitination sites in LKB1.

To date, although there are few reports on the relationship between FBXO22 and tumor, the role of FBXO22 in tumorigenesis remains debatable. FBXO22 has been shown to promote breast cancer cell and hepatocellular carcinoma cell growth^37,41^. However, it was also reported to exert its antimetastatic role in breast cancer and renal cell carcinoma progression^41,42^, and a low level of FBXO22 in tumor tissues predicts a poor outcome in ER-positive/HER2-negative breast cancers^43^. Here, we show that FBXO22 is highly expressed in lung cancer using information not only from available database but also from clinical tumor tissues. Moreover, highly FBXO22 expression has significantly shorter survival times, suggesting its oncogene function. Mechanically, FBXO22 promotes NSCLC cell growth through inhibiting LKB1/AMPK/mTOR signaling pathway, which reinforces the oncogene function of FBXO22 in lung cancer. Further exploring the role of FBXO22 in the initiation and progression of other types of cancer will shed light on the role of FBXO22 in tumorigenesis.

In summary, our study shows that FBXO22 mediates K63-linked polyubiquitination of LKB1, thereby suppresses LKB1 activity and promotes lung cancer cell growth through LKB1/AMPK/mTOR pathway. In addition, FBXO22 is highly expressed in human lung adenocarcinoma and high FBXO22 expression predicts significant poor prognosis. Identification of the FBXO22/LKB1/AMPK/ mTOR axis in this study not only provides great insight into how LKB1 kinase activity is regulated, but also highlights FBXO22 as a potential target for lung cancer therapy.

## Supplementary information

SUPPLEMENTAL MATERIAL
