# Supplementary material for: FBXO22 mediates polyubiquitination and inactivation of LKB1 to promote lung cancer cell growth
Source: Cell Death Dis. 2019 Jun 19;10(7):486. doi: 10.1038/s41419-019-1732-9 (PMC6584689; doi:10.1038/s41419-019-1732-9)
Supplement: Supplementary file 1 — SUPPLEMENTAL MATERIAL [file 41419_2019_1732_MOESM1_ESM.docx]

**SUPPLEMENTAL INFORMATION**

**
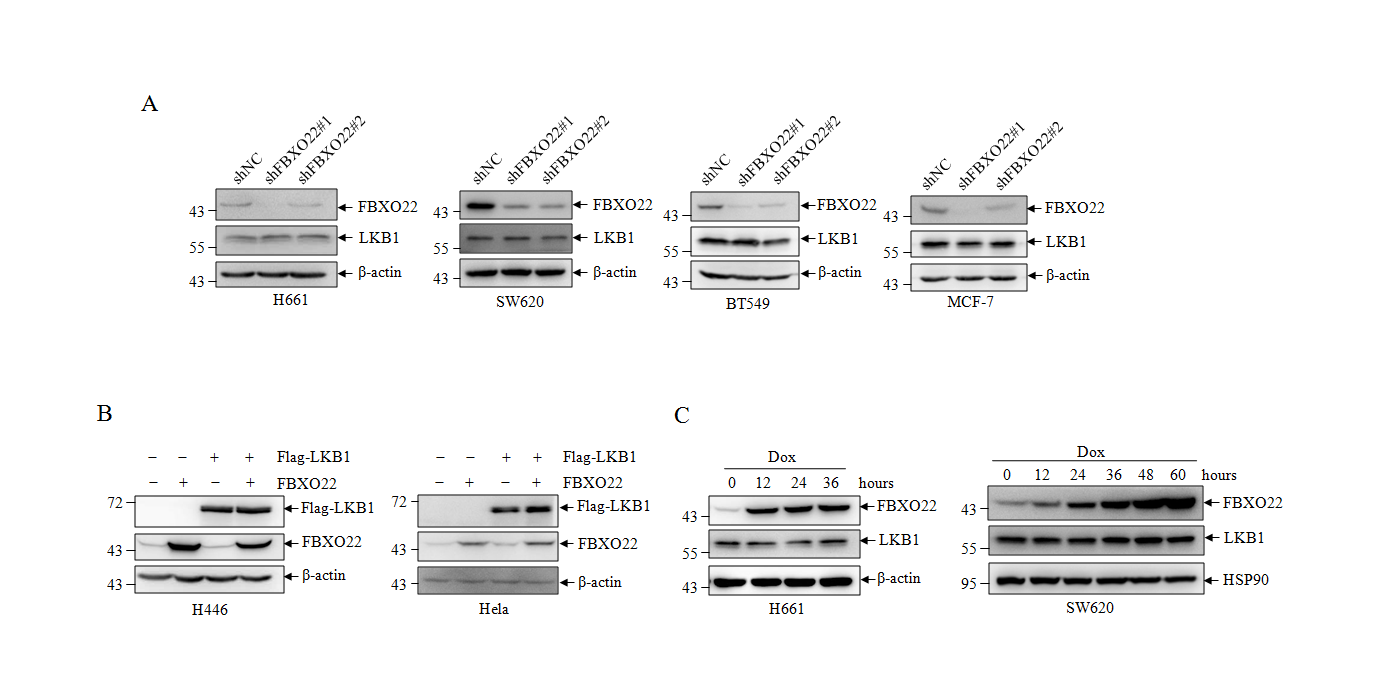
**

Figure S1, related to Figure 4

(A) H661，SW620，BT549 and MCF-7 cells were infected with shNC or shFBXO22，cell lysates were analyzed by Western blots for the indicated proteins.

(B) LKB1-deficient H446 and Hela cells were transfected with Flag-LKB1 and FBXO22, cell lysates were analyzed by western blots for the indicated proteins.

(C) H661 and SW620 cells expressing the doxycycline (Dox) inducible FBXO22 were treated with Dox (100ng/ml) for different time, cell lysates were analyzed by Western blots for the indicated proteins.


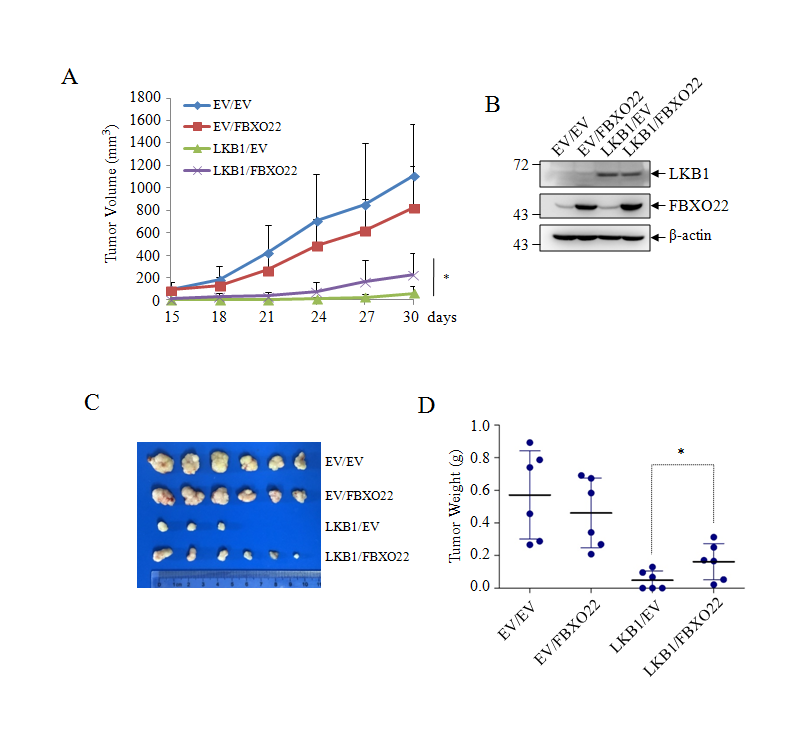


Figure S2, related to Figure 6

(A) EV/EV, EV/FBXO22, LKB1/EV, LKB1/FBXO22 co-transfected Hela cells were subcutaneously injected into nude mice. At least six tumors per condition were analyzed. The tumor growth curve of tumor volume was drawn according the times indicated. Data are presented as mean± S.D. and symbol * indicates p<0.05 between lined groups.

(B) The lysates of tumor tissue from nude mice discribed as (A) were subjected to Western blot to detect the indicated proteins, with β-actin as a loading control.

(C-D) The macroscopic appearances of tumors (C) and the tumor weight (D) were shown at one month after injection. Data are presented as mean± S.D. and symbol *indicates p<0.05 between lined groups.


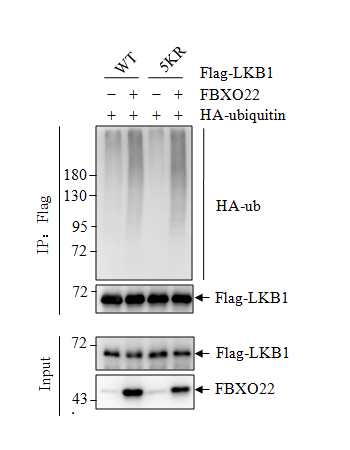


Figure S3 293T cells were transfected with Flag tagged LKB1 or 5KR mutant LKB1 (five K-to-R substitutions at the positions 41, 44, 48, 62 and 64), HA-tagged ubiquitin and FBXO22, then subjected to [denatured](file:///D:\Program%20Files%20(x86)\Dict\7.5.0.0\resultui\dict\?keyword=denatured) co-IP with M2 beads followed by Western blots.

**Supplemental Table S1, related to Figure 1.**

**Information of lung tumor and adjacent Samples**

| **No.** | **Tissues** | **Sex** | **Age** | **FBXO22 scores** |
| --- | --- | --- | --- | --- |
| E05A0013 | Tumor | F | 84 | 4 |
|  | Adjacent normal |  |  | 1 |
| E05A0034 | Tumor | F | 71 | 8 |
|  | Adjacent normal |  |  | 1 |
| E05A0032 | Tumor | F | 72 | 8 |
|  | Adjacent normal |  |  | 1 |
| E05A0036 | Tumor | F | 59 | 4 |
|  | Adjacent normal |  |  | 1 |
| E05A0043 | Tumor | F | 72 | 8 |
|  | Adjacent normal |  |  | 3 |
| E05A0046 | Tumor | F | 66 | 8 |
|  | Adjacent normal |  |  | 4 |
| E05A0060 | Tumor | M | 49 | 8 |
|  | Adjacent normal |  |  | 1 |
| E05A0081 | Tumor | F | 53 | 8 |
|  | Adjacent normal |  |  | 1 |
| E05A0104 | Tumor | M | 74 | 4 |
|  | Adjacent normal |  |  | 1 |
| E05A0123 | Tumor | M | 74 | 8 |
|  | Adjacent normal |  |  | 1 |
| E05A0142 | Tumor | M | 58 | 8 |
|  | Adjacent normal |  |  | 1 |
| E05A0146 | Tumor | M | 30 | 8 |
|  | Adjacent normal |  |  | 4 |
| E05A0149 | Tumor | M | 67 | 4 |
|  | Adjacent normal |  |  | 2 |
| E05A0182 | Tumor | F | 64 | 8 |
|  | Adjacent normal |  |  | 4 |
| E05A0230 | Tumor | F | 52 | 8 |
|  | Adjacent normal |  |  | 4 |
| E05A0201 | Tumor | M | 60 | 4 |
|  | Adjacent normal |  |  | 1 |
| E05A0208 | Tumor | M | 47 | 1 |
|  | Adjacent normal |  |  | 1 |
| E05A0209 | Tumor | M | 65 | 4 |
|  | Adjacent normal |  |  | 1 |
| E05A0210 | Tumor | F | 58 | 12 |
|  | Adjacent normal |  |  | 6 |
| E05A0211 | Tumor | F | 67 | 8 |
|  | Adjacent normal |  |  | 1 |
| E05A0252 | Tumor | F | 50 | 8 |
|  | Adjacent normal |  |  | 1 |
| E05A0253 | Tumor | F | 60 | 8 |
|  | Adjacent normal |  |  | 1 |
| E05A0255 | Tumor | F | 68 | 8 |
|  | Adjacent normal |  |  | 1 |
| E05A0270 | Tumor | M | 55 | 8 |
|  | Adjacent normal |  |  | 1 |
| E05A0272 | Tumor | F | 76 | 12 |
|  | Adjacent normal |  |  | 1 |
| E05A0279 | Tumor | M | 49 | 8 |
|  | Adjacent normal |  |  | 1 |
| E05A0280 | Tumor | M | 73 | 8 |
|  | Adjacent normal |  |  | 1 |
|  |  |  |  |  |
| E05A0282 | Tumor | F | 58 | 8 |
|  | Adjacent normal |  |  | 1 |
| E05A0289 | Tumor | M | 75 | 8 |
|  | Adjacent normal |  |  | 1 |
| E05A0310 | Tumor | F | 69 | 4 |
|  | Adjacent normal |  |  | 1 |
| E05A0317 | Tumor | F | 57 | 8 |
|  | Adjacent normal |  |  | 4 |
| E05A0318 | Tumor | M | 75 | 8 |
|  | Adjacent normal |  |  | 2 |
| E05A0321 | Tumor | F | 52 | 4 |
|  | Adjacent normal |  |  | 1 |
| E05A0322 | Tumor |  |  | 4 |
|  | Adjacent normal |  |  | 1 |
| E05A0332 | Tumor | M | 55 | 4 |
|  | Adjacent normal |  |  | 1 |
| E05A0333 | Tumor | M | 65 | 12 |
|  | Adjacent normal |  |  | 2 |
| E05A0349 | Tumor | M | 42 | 4 |
|  | Adjacent normal |  |  | 1 |
| E05A0353 | Tumor | M | 60 | 6 |
|  | Adjacent normal |  |  | 1 |
| E05A0355 | Tumor | M | 53 | 4 |
|  | Adjacent normal |  |  | 1 |
| E05A0359 | Tumor | M | 66 | 8 |
|  | Adjacent normal |  |  | 1 |
| E05A0363 | Tumor | M | 68 | 4 |
|  | Adjacent normal |  |  | 1 |
|  |  |  |  |  |
| E05A0364 | Tumor | F | 65 | 8 |
|  | Adjacent normal |  |  | 1 |
| E05A0392 | Tumor | F | 57 | 4 |
|  | Adjacent normal |  |  | 1 |
| E05A0399 | Tumor | F | 51 | 12 |
|  | Adjacent normal |  |  | 1 |
| E05A0403 | Tumor | M | 64 | 8 |
|  | Adjacent normal |  |  | 3 |
| E05A0426 | Tumor | M | 71 | 4 |
|  | Adjacent normal |  |  | 1 |
| E05A0448 | Tumor | F | 60 | 8 |
|  | Adjacent normal |  |  | 1 |
| E05A0449 | Tumor | M | 61 | 9 |
|  | Adjacent normal |  |  | 2 |
| E05A0456 | Tumor | F | 62 | 6 |
|  | Adjacent normal |  |  | 1 |
| E05A0463 | Tumor | F | 58 | 3 |
|  | Adjacent normal |  |  | 1 |
| E05A0471 | Tumor | M | 63 | 4 |
|  | Adjacent normal |  |  | 1 |
| E05A0475 | Tumor | M | 63 | 2 |
|  | Adjacent normal |  |  | 1 |
| E05A0482 | Tumor | M | 61 | 4 |
|  | Adjacent normal |  |  | 6 |
| E05A0486 | Tumor | F | 81 | 8 |
|  | Adjacent normal |  |  | 1 |
| E05A0502 | Tumor | M | 61 | 2 |
|  | Adjacent normal |  |  | 1 |
|  |  |  |  |  |
| E05A0663 | Tumor | M | 84 | 2 |
|  | Adjacent normal |  |  | 1 |
| E05A0519 | Tumor | M | 65 | 12 |
|  | Adjacent normal |  |  | 1 |
| E05A0537 | Tumor | M | 53 | 8 |
|  | Adjacent normal |  |  | 1 |
| E05A0520 | Tumor | M | 74 | 8 |
|  | Adjacent normal |  |  | 1 |
| E05A0523 | Tumor | M | 64 | 4 |
|  | Adjacent normal |  |  | 2 |
| E05A0546 | Tumor | F | 73 | 4 |
|  | Adjacent normal |  |  | 1 |
| E05A0550 | Tumor | M | 52 | 8 |
|  | Adjacent normal |  |  | 1 |
| E05A0580 | Tumor | M | 44 | 6 |
|  | Adjacent normal |  |  | 1 |
| E05A0565 | Tumor | M | 55 | 8 |
|  | Adjacent normal |  |  | 1 |
| E05A0584 | Tumor | F | 50 | 4 |
|  | Adjacent normal |  |  | 1 |
| E05A0622 | Tumor | M | 78 | 8 |
|  | Adjacent normal |  |  | 0 |
| E05A0592 | Tumor | F | 60 | 4 |
|  | Adjacent normal |  |  | 1 |
| E05A0595 | Tumor | F | 54 | 4 |
|  | Adjacent normal |  |  | 1 |
| E05A0596 | Tumor | F | 48 | 8 |
|  | Adjacent normal |  |  | 1 |
|  |  |  |  |  |
| E05A0616 | Tumor | M | 59 | 4 |
|  | Adjacent normal |  |  | 1 |
| E05A0617 | Tumor | M | 48 | 4 |
|  | Adjacent normal |  |  | 1 |
| E05A0626 | Tumor | M | 71 | 12 |
|  | Adjacent normal |  |  | 1 |
| E05A0627 | Tumor | M | 59 | 8 |
|  | Adjacent normal |  |  | 1 |
| E05A0633 | Tumor | M | 58 | 2 |
|  | Adjacent normal |  |  | 0 |
| E05A0639 | Tumor | F | 56 | 12 |
|  | Adjacent normal |  |  | 4 |
| E05A0640 | Tumor | F | 53 | 8 |
|  | Adjacent normal |  |  | 0 |
| E05A0648 | Tumor | F | 62 | 8 |
|  | Adjacent normal |  |  | 1 |
| E05A0659 | Tumor | M | 72 | 12 |
|  | Adjacent normal |  |  | 1 |
| E05A0671 | Tumor | M | 61 | 12 |
|  | Adjacent normal |  |  | 1 |
| E05A0682 | Tumor | F | 65 | 12 |
|  | Adjacent normal |  |  | 1 |
| E05A0677 | Tumor | F | 67 | 8 |
|  | Adjacent normal |  |  | 2 |
| E05A0699 | Tumor | M | 65 | 12 |
|  | Adjacent normal |  |  | 1 |
| E05A0702 | Tumor | M | 65 | 8 |
|  | Adjacent normal |  |  | 1 |
|  |  |  |  |  |
| E05A0703 | Tumor | F | 77 | 3 |
|  | Adjacent normal |  |  | 1 |
